# Using Impedance Measurements to Characterize Surface Modified with Gold Nanoparticles

**DOI:** 10.3390/s17092141

**Published:** 2017-09-18

**Authors:** Scott MacKay, Gaser N. Abdelrasoul, Marcus Tamura, Donghai Lin, Zhimin Yan, Jie Chen

**Affiliations:** 1 Department of Electrical and Computer Engineering, University of Alberta, Edmonton, AB T6G 1H9, Canada; samackay@ualberta.ca (S.M.); gasernag@ualberta.ca (G.N.A.); matamura@ualberta.ca (M.T.); donghai1@ualberta.ca (D.L.); 2 National Institute for Nanotechnology, National Research Council, Edmonton, AB T6G 2M9, Canada; Zhimin.Yan@nrc-cnrc.gc.ca; 3 Department of Biomedical Engineering, University of Alberta, Edmonton, AB T6G 2V2, Canada

**Keywords:** impedance-based biosensor, gold nanoparticles, point-of-care biosensor, impedance measurements, surface characterization

## Abstract

With the increased practice of preventative healthcare to help reduce costs worldwide, sensor technology improvement is vital to patient care. Point-of-care (POC) diagnostics can reduce time and lower labor in testing, and can effectively avoid transporting costs because of portable designs. Label-free detection allows for greater versatility in the detection of biological molecules. Here, we describe the use of an impedance-based POC biosensor that can detect changes in the surface modification of a micro-fabricated chip using impedance spectroscopy. Gold nanoparticles (GNPs) have been employed to evaluate the sensing ability of our new chip using impedance measurements. Furthermore, we used impedance measurements to monitor surface functionalization progress on the sensor’s interdigitated electrodes (IDEs). Electrodes made from aluminum and gold were employed and the results were analyzed to compare the impact of electrode material. GNPs coated with mercaptoundecanoic acid were also used as a model of biomolecules to greatly enhance chemical affinity to the silicon substrate. The portable sensor can be used as an alternative technology to ELISA (enzyme-linked immunosorbent assays) and polymerase chain reaction (PCR)-based techniques. This system has advantages over PCR and ELISA both in the amount of time required for testing and the ease of use of our sensor. With other techniques, larger, expensive equipment must be utilized in a lab environment, and procedures have to be carried out by trained professionals. The simplicity of our sensor system can lead to an automated and portable sensing system.

## 1. Introduction

Biosensors are a popular research area because of their vast potential for improving healthcare. The ability to test for a specific biomarker can help detect diseases such as cancer while they are still localized [1,2]. While there are technologies in existence that can perform such tasks, they often require samples to be tested at a laboratory dedicated to this purpose. Both transporting and testing the sample can be expensive and time consuming, making a point-of-care design ideal in terms of time and cost efficiency [3,4]. Various bio-sensing techniques have been proposed using optical [5,6,7], electrical [8,9], and mechanical [10,11,12] detection methods. Compared to optical methods of detection, impedance tests are easier to use and more versatile. The equipment often required for optical detection is too large, complex, and expensive for portable point-of-care testing. However, optical methods tend to achieve high sensitivity, which is why they are commonly used [13]. Mechanical methods, on the other hand, can yield specific and sensitive results, but they are much more prone to inaccuracies due to temperature and pH changes [14].

**Figure 1:**
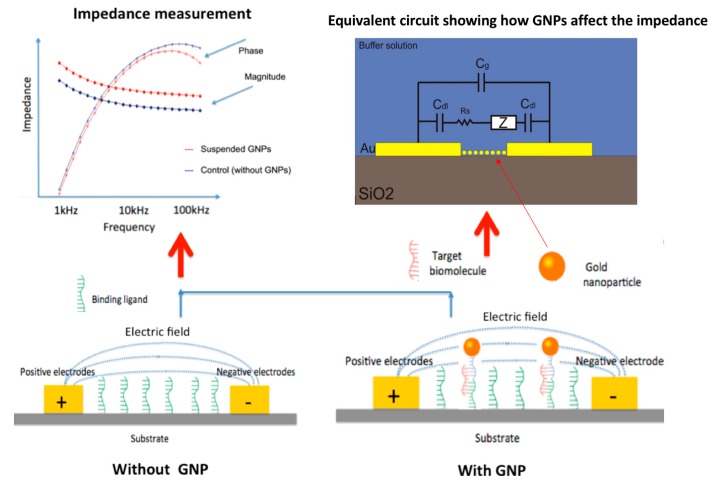
Gold nanoparticles bound by target biomolecules disrupt the electric field between electrodes, causing a change in impedance. The insert shows the equivalent circuit of the interdigitated electrodes (IDEs) sensor system with bound gold nanoparticles (GNPs).

Impedance-based detection methods have been shown to have a lower maximum sensitivity than other techniques [15]. However, we can enhance the sensitivity of the impedance measurements by using gold nanoparticles (GNPs) [16]. Such a design yields more precise results. The mechanism by which GNPs affect the impedance of the system can be represented using the equivalent circuit shown in Figure 1. This circuit is a combination of the double layer capacitance of the electrode digits (C_dl_), the physical capacitance of the electrode (C_g_), the resistance of the surrounding buffer solution (R_s_), and the impedance introduced by the bound nanoparticles (Z). Since this is a non-faradic system that does not depend on electron transfer from electrochemical reactions, complex elements, such as electron transfer resistance and mass transfer resistance, are not required [17]. The GNPs provide a change in impedance through the phenomenon of double layer capacitance. Charges in the solution surrounding the nanoparticles build up around the oppositely charged surface of the GNP. This forms two layers of charge (double layer). The build-up of charge closely resembles the effects of a capacitor. Consequently, the GNPs provide a capacitive change when bound to the surface between electrodes. Nanoparticles have been used in a variety of biological applications, such as drug and gene delivery, cancer therapy, and protein detection [18,19,20]. GNPs have been shown to be highly adjustable in their modification [15,21], and assorted methods for binding GNPs using aptamers exist [22,23]. An aptamer is a biological molecule that can bind to a specific substance with extremely high specificity. They are typically constructed from oligonucleotides or peptides.

**Figure 2:**
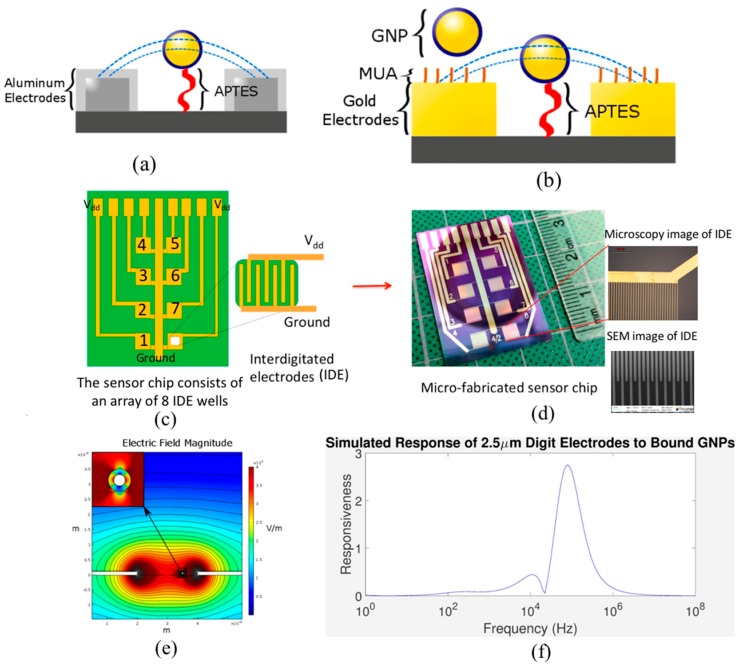
(**a**) Schematic of aluminum electrodes with oxide coating and electric field lines. The oxide layer acts as a capacitor as electric field lines pass through it. (**b**) Illustration of how the 11-Mercaptoundecanoic acid (MUA) creates a layer over the gold IDEs. Note that this layer can also prevent GNPs from settling on the surface of the electrodes via metallic bonding (Note: both of these are not to scale.). (**c**,**d**) The impedance-based sensor chip design (schematic vs. real chip). The insert shows the design of IDEs. The eight wells are depicted as the squares, which are IDEs. Each well connects to its own electrode connector pad. (**e**) COMSOL simulations of IDEs with bound gold nanoparticles. The simulation layout with the electric field magnitude shown. (**f**) A graph showing the impedance magnitude sensitivity of IDEs to GNPs bound to different positions between electrode digits as a function of frequency, where “responsiveness” is the percent difference in the impedance phase between the electrode with and without bound nanoparticles.

Microfabricated chips with interdigitated electrodes (IDEs) were used to characterize surface modification with GNPs (Figure 2). IDE sensing chips were created to have 3 mm × 3 mm squares of IDEs on a silicon dioxide substrate (refer to Figure 2c,d). The electrodes were created using standard photolithographic microfabrication techniques to create 2 μm of spacing between electrode digits that are 4 μm wide. Many other groups have created IDEs, but they typically use bigger dimensions as these are easier to microfabricate [24,25,26,27]. Some other groups have developed structures that are more complex to enhance sensitivity [28]. Furthermore, these structures are more difficult to microfabricate. Our design uses smaller electrode width, height and spacing dimensions than other groups. The smaller dimensions can also enhance sensitivity. Evidence has shown that the smaller the spacing, the more sensitive the impedance is to the presence of chemical species in between IDEs [29]. Some example results from these simulations using COMSOL Multiphysics (version 5.3, COMSOL Inc. Burlington, MA, USA) are shown in Figure 2e,f. These results show the layout of the simulation along with the associated electric field distribution, and the simulated sensitivity of IDEs to bound nanoparticles. Results show a specific distribution of sensitivities for nanoparticles bound to IDEs as well as the predicted behavior and frequency range of interest for impedance measurements. The purpose of experiments presented here are as proof-of-concept experiments of the detection principle shown by the simulations. The change in impedance should rely only on the binding of nanoparticles, so directly binding GNPs chemically to IDEs should result in the same effect as the simulations. The simulation also proves the efficacy of this method to be used with molecular recognition elements for future biosensing applications.

Tests were performed on chips made of either aluminum or gold electrodes (Figure 2a,b). Aluminum electrodes have the benefit of having a naturally occurring aluminum oxide layer. Aluminum oxide is an excellent dielectric increasing the capacitive character of our design. This is preferable because the change in capacitance from the addition of GNPs is the primary cause of impedance change detected. Gold electrodes are far more chemically stable. Its noble metal features allow for less corrosion and unwanted chemical reactions in buffer and salt solutions. Gold does not react with most biological molecules making it useful for sensing these types of molecules. However, the weak adhesion affinity of the gold on silicon dioxide substrate makes its microfabrication much more difficult and expensive. While aluminum electrodes are easier and more reliable to fabricate, gold electrodes are much more resistant to corrosion and have greater biocompatibility. Another benefit for gold electrodes is their affinity for molecules with thiol groups. This allows for surface modifications of the electrodes themselves after microfabrication. The feasibility of the electrode’s surface for chemical modification from both of the gold and silicon dioxide areas make it a good candidate for immobilizing several biomarkers by engineering the surface chemistry.

Modifications of the electrode surfaces using a self-assembled monolayer were also investigated [30]. It has been shown to reduce corrosion, [31,32], and it was hypothesized that it would mimic the effect of the oxide layer on aluminum electrodes (Figure 2a). In addition, a monolayer over the electrodes can prevent other large debris from non-specifically binding to the gold IDEs. This is especially important for the development of this biosensor as real biological samples may contain bacteria or other impurities. For instance, in the detection of a protein as an analyte, the protein can be covalently attached to the gold surface through the thiol group of its cysteine and/or glutathione amino acid moieties. These can stick to the electrode surface and can interfere with impedance readings. Since the purpose of this biosensor is for use in the field or in a clinic, protection of the electrodes from dust-filled environments is paramount. Extensive filtration can prevent this issue, but may not be feasible outside of the laboratory environment. Passivation of the electrode surface was accomplished by modifying the gold IDEs with 11-Mercaptoundecanoic acid (MUA) via its thiol group (Figure 2b). Other researchers have performed similar procedures [32,33]. This type of modification is well-defined and can be used to bind certain molecules to a surface [34,35]. Here, the experimental results performed on chips with both aluminum and gold electrodes that have been chemically activated to covalently bind with surface modified GNPs are shown and discussed. Firstly, the IDEs were coated with (3-Aminopropyl)triethoxysilane (APTES) at different concentrations. The GNPs were coated with MUA facilitating the binding with chemical modified electrode. At each APTES concentration, GNPs were bound using 1-Ethyl-3-(3-dimethylaminopropyl)carbodiimide/*N*-Hydroxysuccinimide (EDC/NHS) chemistry as a model for biological binding. This is to investigate the effects of the GNPs solely and prove the theoretical concept previously reported. Due to the versatility of GNPs modification, the sensor should allow for the detection of a variety of biological molecules. Modifications have been characterized through impedance spectroscopy and with an atomic force microscope (AFM). These results are compared with simulation results that have been performed using the design of the chip, which have been previously reported [16,36]. COMSOL Multiphysics software was used to create simulations of IDEs with a range of dimensions, buffers, and nanoparticle positions. The position of the nanoparticle in relation to the IDE was also simulated for the distance from the surface of the electrode. These simulation results showed that there was no significant change in sensitivity based on the separating distance between the nanoparticle and the IDEs substrate as long as it never exceeds the height of the electrode digits. This result is particularly relevant for future tests involving different recognition elements. DNA, proteins, and antibodies can have very different sizes, so nanoparticles modified with these biomolecules would bind to the electrodes at different separating distance. The results of these simulations show that, regardless of the recognition element used, the sensitivity should remain the same.

## 2. Methods

### 2.1. Basic Impedance Tests

A polydimethylsiloxane (PDMS) well covering was placed on top of the chip, which provided a well to hold a solution of electrolyte over each well. PDMS well coverings were constructed to have a perfectly flat bottom surface to ensure a water-tight seal on the microfabricated electrodes. Then, 50 μL of 150 μM KCl solution were placed onto each well as an electrolyte. KCl has a neutral pH and should not interfere much with biological reagents because K+ and Cl− are weak oxidizing and reducing agents, respectively. This relatively simple and stable solution does not react readily with GNPs, which makes it a useful electrolyte for our purposes [37]. The concentration of KCl carried a current between electrodes as an electrolyte and lowered the effect of extraneous substances from changing the ionic strength of the solution such as carbon dioxide. Carbon dioxide dissolving from air and becoming ionized carbonic acid could increase the electrolyte’s ionic strength. Therefore, the ionic strength needed to be high enough to limit the effect this phenomenon may have. However, if the ionic strength of the solution is too high, its impedance overshadows any changes in surface modification.

This sensor design is intended to be used as a biosensor in future applications. In general, biological samples that would be tested have much higher ion concentrations than our measurement buffer used in the current studies. This factor has to be taken into consideration when designing the sensor. Biological conditions have ionic concentrations that are likely too high for this detection to work properly. However, when biological samples are used, the chemical binding part of the sensing is the only part that requires these concentrations. After exposure to the biological sample (and resulting biological conditions), the sample is removed and a “measurement buffer” with the appropriate ionic concentration is added for the impedance measurement to take place.

Impedance measurements were done using a voltage amplitude of 10 mV, and a frequency sweep of 10 kHz to 1 MHz for the aluminum electrodes and a larger sweep of 1 kHz to 1 MHz for gold electrodes. Each frequency was tested five times and their average was used. Impedance spectroscopy was carried out using an electrochemical measurement station (SP-200, BioLogic Inc., Seyssinet-Pariset, France). After testing, chips were rinsed with Milli-Q water, sonicated in Milli-Q water and then washed using ethanol.

### 2.2. Synthesis and Surface Functionalization of GNPs

The synthesis of the GNPs followed the Turkevich-Frens method using trisodium citrate dihydrate (TSC) (Sigma-Aldrich, Oakville, ON, Canada) as both a reducing and dispersion agent. Hydrogentetrachloroaurate trihydrate (HAuCl4.3H2O) (Sigma-Aldrich, 99.9%) was employed as a gold precursor, where the synthesis and the nanoparticle washing were performed as previously reported [38]. The prepared NPs were characterized by transmission electron microscopy (Philips 410 TEM, Eindhoven, The Netherlands), inductive plasma couple combined with atomic emission spectroscopy (Thermo Scientific iCAP 7000 Series, Burlington, ON, Canada) and optical spectroscopy (Agilent 8453 UV-vis Spectrophotometer, Hampton, ON, Canada).

The surface functionalization of the GNPs was carried out as follows. To a 5 mL dispersion of GNPs with an estimated concentration 0.22 μM, a 5 mL aqueous solution consisting of 2 mM Poly(ethylene glycol) methyl ether thiol Molecular Weight 800 (mPEG-SH, Sigma-Aldrich) and 2 mM of MUA (Sigma-Aldrich) was added dropwise. The concentration of MUA was selected to be 20% (*v*/*v*). The reaction solution was stirred overnight to ensure sufficient functionalization of the nanoparticles. Then, the MUA-GNPs were collected by centrifugation at 13,000 rpm for 10 min. The MUA-GNPs were washed by redispersing the NPs plate in Milli-Q water. The last two steps were repeated two more times to completely remove the unbound thiol compounds. The nanoparticles surface’s functionalization was evaluated by zeta potential measurement (Zetasizer Malvern, Malvern, UK).

### 2.3. APTES Modification

**Figure 3:**
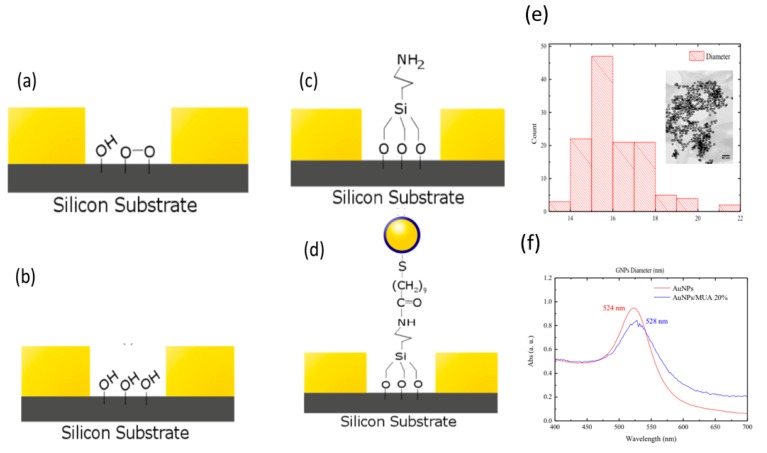
(**a**) Silicon IDE chip after microfabrication. Silicon dioxide substrate has reacted with oxygen in the air, but not always in the same fashion; (**b**) oxygen plasma removes the silicon oxide layer and it is replaced with hydroxy groups; (**c**) hydroxy groups are used as binding sites for the (3-Aminopropyl)triethoxysilane (APTES) which have a free amine group; (**d**) GNPs are bound to the APTES using 1-Ethyl-3-(3-dimethylaminopropyl)carbodiimide/*N*-Hydroxysuccinimide (EDC/NHS) chemistry; (**e**) the histogram of the GNPs’ size distribution, and the insert shows the TEM image of GNPs dispersion; (**f**) the UV-Vis absorption spectra of the GNPs before and after MUA surface coating.* Note: These figures are not to scale. The electrodes are much taller than this image suggests.

Before treating the IDEs with APTES, oxygen plasma cleaning (Figure 3a–d) was used to clean and activate the silicon surface. APTES ethanolic solutions of different concentrations (2%, 5%, 7% and 10%) were freshly prepared. APTES solutions were individually drop casted on the top of the IDEs and incubated for 2 h. Afterwards, the APTES solution was removed and the IDEs were washed several times using absolute ethanol. After rinsing and sonication in both Milli-Q water and ethanol, impedance tests were performed.

### 2.4. MUA-GNPs/APTES Modified IDEs Binding

The binding of the MUA-GNPs to the APTES-IDEs was performed using EDC/NHS bioconjugation chemistry [39]. The carboxylic group at the nanoparticles surface was activated by addition of 30 mM *N*-Hydroxysuccinimide (NHS (Sigma-Aldrich, 98%), and 15 mM *N*-(3-Dimentylaminopropyl)-*N′*-ethylcarbodiimide (EDC, Sigma-Aldrich) in 4-Morpholine-ethanesulfonic acid (MES) buffer (Sigma-Aldrich), to MUA-GNPs dispersion. A PDMS template cover with 8 wells was fixed over the chip, where each well set directly over each IDEs. Later, 50 μL of the activated MUA-GNPs were added in each well and the IDEs were incubated overnight to complete the binding. The NPs solution was discarded from each well and all IDEs were washed several times with Milli-Q water removing unbound NPs. The chip was then rinsed and sonicated one last time, and impedance measurements were performed.

### 2.5. Gold Electrode Surface Modification

The thiol groups on the MUA is bound to the gold IDEs (Figure 2b). Thiol groups have a high affinity for gold [40,41,42]. A 50 μL solution of 1 mM MUA was placed on each well for overnight to allow for adsorption of the MUA to the surface of the electrodes. This creates a layer of a long-chain alkane with a carboxy-functional group on the end. This functional group helps to prevent molecules from chemically binding to the surface. After this was accomplished, the electrode was modified by APTES and GNPs in the same way as above.

## 3. Results

### 3.1. Gold Nanoparticles Synthesis and Surface Modification

The GNPs prepared by the Turkevich–Frens method [43] showed monodispersion with average diameter of 16 ± 2 nm as estimated from measuring the diameter of 125 nanoparticles (refer to the insert of Figure 3e). The UV-Vis absorption of the GNPs exhibited a typical plasmonic absorption with maximum at 524 nm (refer to Figure 3f) [44]. This maximum was red shifted to 528 nm after modifying the NPs surface by MUA and Poly(ethylene glycol) methyl ether thiol(mPEG-SH) indicating the efficient surface coating. The surface coating of the GNPs was further confirmed using dynamic light scattering (DLS) and ζ-potential measurements. After coating the GNPs with MUA and mPEG-SH, the hydrodynamic size decreased from 110 ± 15 nm to 55 ± 5 nm, and the ζ-potential decreased from −23 mV to −15 mV. The alteration in the hydrodynamic size might be attributed to the nature of the interaction of the surface modifier with the surround monolayer of the solvent. However, the decrease in the NPs surface charge might be due to the attachment of the mPEG-SH molecules to the surface of the NPs. The use of mPEG-SH was found enhancing the dispersion stability of the GNPs in aqueous medium after surface modification. This is due to the hydrophilic nature of mPEG-SH.

The hydrodynamic size of the nanoparticles represents not only the size of the nanoparticles with the coating layer but also the interfacial zone consists of the first adjacent layers of the solvent surrounding the nanoparticle itself. This interfacial zone is defined by the nature and capacity of the coating molecular layer to interact with the surrounding solvent molecules, which, in turn, depends on the functional groups anchored to the coating molecules. In the case of MUA, there is only one carboxylic group attached by an aliphatic chain of eleven carbon atoms to the surface of GNPs. The interaction of the surrounding solvent molecules with the GNPs occurred through only one carboxylic group. On the other hand, the sodium citrate surfactant molecule has three carboxylic groups and one hydroxyl group. One of these carboxylic groups attaches directly to the nanoparticle surface, increasing its dispersion stability, while the rest are interacting with the surrounding solvent molecules through hydrogen bonding. This could increase the number of water molecules attracted the GNPs, and, consequently, increases the hydrodynamic size. The functionalization with mPEG-SH decreases the number of hydrogen bonding sites on the GNP, which decreases the number of water molecules that are hydrogen-bond to the GNP. This decreases the hydrodynamic size.

### 3.2. Effect of Oxygen Plasma on Electrodes and Impedance Measurements

From Table 1 and Table 2, we can clearly see a lower average impedance in all oxygen plasma results. The change in the surface chemistry due to oxygen plasma cleaning is also noticed in the lower coefficient of variation across all frequencies. The oxygen plasma cleaning (refer to Figure 3a–d) was used to clean the surface of the electrode and activate the silicon dioxide substrate for coating with APTES by initiating hydroxyl groups. The hydroxyl groups affected the average impedance of the IDE.

### 3.3. Impedance Measurements of GNPs on Gold and Aluminum IDEs

**Figure 4:**
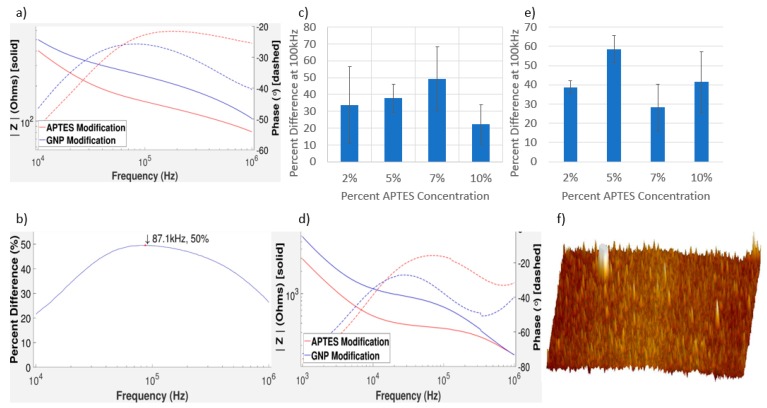
(**a**) The Bode graph of the impedance measurement after APTES and after GNP modification on a gold IDE; (**b**) the graph of the percent difference between the impedance magnitudes of the GNP modification with respect to the APTES modification. The red dot indicates the maximum change in impedance; (**c**) the APTES concentration was varied and graphed against the percent difference at 100 kHz of each respective test; (**d**) impedance measurements after APTES modification and GNP modification on an aluminum IDE; (**e**) the APTES concentration was varied and graphed against the percent difference at 100 kHz of each respective test; (**f**) a 3D image of the chip surface after GNP modification. The many small spikes are GNPs. * Note: in the impedance plots, the solid lines are for the magnitude response while the dot lines are for the phase response.

Surface modification clearly has an effect on the impedance from Figure 4. Modification with GNPs created an increase in impedance due to double layer capacitance. The surface bound GNPs act as capacitors because they form a double layer around their surface [45]. This increase in capacitive character of the chip is noticed in the decrease in the phase of the graph. The greatest change in impedance can be found near 100 kHz. In regards to detecting changes in the surface modification, the middle frequency range is ideal. This is because lower frequencies can be more prone to changes in the variation between measurements. At high frequencies, all measurements appear to converge no matter the type or amount of surface modification. The APTES serves as a binding site for the GNPs. Concentrations of the APTES solutions were varied between wells as percentages of the stock solution in order to understand the effects of surface coverage on impedance. Theoretically, we should see an increasing trend in Figure 4c. This is because, the more APTES that is added to the chip, the more binding sites are available to the GNPs. This, in turn, leads to more GNPs on the surface, which can create a larger change impedance. Instead, from our experimental results (Figure 4e), we see impedance increases from 2 to 5% and 7 to 10% APTES concentration, with a sharp impedance drop when APTES concentration increases from 5 to 7%. The sharp drop is likely due to an oversaturation of APTES between the electrodes.

The error bars in Figure 4c come from the standard deviation of multiple tests taken with the same APTES concentrations. Errors between results could be due to a number of factors. Electrode chips were manufactured using wet etching techniques that can have errors in fabrication. All microfabrication techniques have some errors associated with the area of fabrication. This causes different chips to have slightly different dimensions, in different areas of the chip.

Compared to simulation results of a virtual chip with the same dimensions and electrode material, an increase due to the surface modification with GNPs matches our experimental results [16]. Simulation results also predicted a peak percent change at a specific frequency, which we see in Figure 4b. It is important to recognize that, compared to some electrochemical impedance spectroscopy designs, our impedance measurements take place after surface modification, not during a reduction-oxidation reaction between a molecule and the surfactant. This allows for the optimization of adsorption of GNPs or other biological molecules to the surface to improve precision and accuracy.

### 3.4. Impedance Measurements of GNPs on Gold IDEs

In experimentation, a larger frequency range of the gold electrodes was taken, in order to encompass the graph shape. This allows us to compare the shapes of the graphs more easily. The fact that we need to do this suggests that the impedance of the gold electrodes is more resilient to changes in the frequency. Since the impedance of a capacitor is related to the frequency and the impedance of a resistor is not, it suggests that the gold is more resistive than their aluminum counterparts. The addition of GNPs to the surface leads to a higher impedance and increases the capacitive character of the chip. We can tell the chip is more capacitive from the decrease in phase. Comparing Figure 4a–d, note that aluminum and gold have very similar shapes and trends, despite the difference in their IDE materials. Simulations predicted that the differences between gold and aluminum electrodes would not be great [16]. However, there was a difference in how closely the change in impedance correlated with the APTES concentration. For gold electrodes, the graph of percent difference versus APTES concentration has again shown that the more GNPs, the greater the change in impedance. This trend could allow for the construction of a concentration curve, which could be used to predict the concentration of GNPs added to the surface.

The error in the impedance tests of gold electrode chips is in general higher than the error for the aluminum electrode chips. As previously mentioned, microfabrication can yield chips with slightly different dimensions. Gold is more difficult to work with in regards to microfabrication because of its natural chemical stability. The chemicals required to etch gold tend to be corrosive enough to destroy other structures on the chip that should be kept fixed. Inevitably, gold electrode chips are slightly over-etched, leading to more spacing between the electrodes. In Figure 4e, the 2% APTES results have significantly more error because they were more prone to clumping. The GNP concentration in solution was kept high to ensure all binding sites were filled on the surface. The 2% APTES tests had less binding sites on the surface and therefore more GNPs that could non-specifically bind and cluster on the gold electrodes. This affected the gold IDEs because the aluminium had the oxide layer to prevent any metallic bonding.

### 3.5. Characterizing the Distribution of GNPs on IDEs

The parabolic shape shown in Figure 4b allows us to easily optimize the frequency that we measure. The average maximum frequency from our results is around 85.7 kHz. This contrasts with simulation predictions, where the peak frequency was on the order of 30 kHz [16]. Optimization of the frequency is important as it could lead to higher sensitivity. This peak frequency could shift depending on how GNPs are bound to the surface. Therefore, the peak frequency could depend on the target biomolecule of interest. There is a correlation between APTES concentration and impedance change. This is because, when the APTES concentration is changed in solution, it changes the surface coverage of the binding sites available for GNP binding. This directly affects how many GNPs can bind to the surface, and thus we can see the effect the amount of GNPs has on the surface of the chip. To validate this finding, atomic force microscopy (AFM) was also used to check the distribution of GNPs on the surface of the sensor chip.

Based on Figure 4f, we can clearly see that the GNPs are on the surface as the validation of our impedance results. With the exception of the large pillar of GNPs at the top left of Figure 4f, the distribution of GNPs is fairly uniform with no localized pitting. Non-uniformities in the GNP distribution can theoretically influence the impedance of the surface. However, as long as the surface has little clumping and there are a large number of interdigitated electrodes, this effect is often insignificant. The clumping of GNPs on the electrodes is something that should be avoided, as it can sometimes create unpredicted impedance results.

### 3.6. Oversaturation of APTES

**Figure 5:**
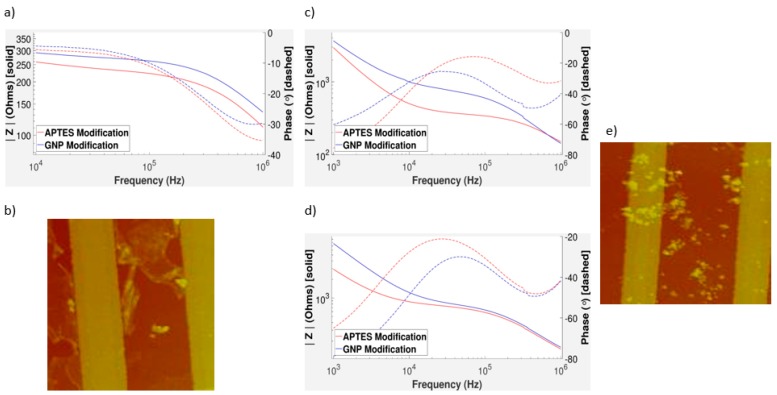
(**a**) The impedance measurement graph for another GNP test with a higher concentration of APTES; (**b**) the Atomic Force Microscopy (AFM) measurement from this well; (**c**) impedance measurements of a different typical surface modification of APTES (2% concentration); (**d**) an impedance measurement on IDES that had a higher than normal aggregation of GNPs in some areas; (**e**) an image taken on this electrode with an AFM (10 μm × 10 μm area shown).

Oversaturation of APTES on the surface can interfere with GNP binding. Figure 5 shows the effects of higher concentrations of APTES being added to the surface. Based on the impedance measurement graph, there is little to no change from the GNP modification, and the graph has a very different shape in both the phase and the magnitude. From this graph, it indicates that the chip is short-circuited because of APTES accumulated between IDEs, showing a low impedance and near zero phase. Figure 5b visibly shows the higher APTES concentrations forming three-dimensional complexes, rather than a uniform surface coverage [46]. GNP binding likely did not occur because the upper layers of APTES blocked the binding sites, which is supported from the diminutive change in the impedance measurement graph. From Figure 5c, the decrease in impedance magnitude after a typical APTES surface modification suggests that APTES has some semiconductor properties when adsorbed onto a surface. The addition of APTES to the surface of the electrode leads to a lower impedance in the gold IDEs as well as the aluminum IDEs. This decrease occurs over all frequencies, but tends to affect the middle frequencies more heavily than the extremes. This again validates the theory that changes in surface coating is not the dominant factor of the impedance at extreme frequencies.

In Figure 4c,e, it is shown that there is a sharp drop at higher concentrations of APTES. Oversaturation is a likely cause of this for a several reasons. Figure 5a shows an extreme case of oversaturation of APTES causing a lower change in impedance between APTES and GNP modification, which matches Figure 4c,e. Aluminum electrode chip results showed a much larger error in the average percent difference at higher APTES concentrations. The formation of 3D layers over a surface is not particularly well-defined, as there are many different geometries possible. It is plausible that different geometries would give different impedance magnitudes as more or less binding sites are covered with APTES. This would make the changes in impedance less predictable; however, some binding sites would still remain open allowing for an overall increase in impedance change. In Figure 4c, there is still an increase from 7 to 10% APTES. Even though the 10% APTES tests were more likely to oversaturate, this effect was random and did not affect every experiment. In addition, 10% APTES tests that did not form three-dimensional complexes on the surface had higher impedance changes because there were more binding sites. However, this was infrequent, which led to increased error for these tests. The oversaturation effect made itself more apparent at a higher APTES concentration in aluminum than in the gold. The gold electrodes are, in general, more over-etched, so there is more space between the electrodes. This means it can hold more APTES on its surface before becoming oversaturated, which is shown in our aluminum versus gold results.

The oversaturation of APTES gives unpredictability and a lower detectable change. Thus, it is important that the silicon substrate is not oversaturated with APTES. It is for this reason that 7% concentration was chosen as our maximum usable concentration for gold electrodes. While higher concentrations of APTES surface coverage could theoretically yield higher changes in impedance magnitude, they also carry more risk of short-circuiting and three-dimensional complexes on the surface.

### 3.7. Effect of Clumped GNPs and MUA Surface Modification

GNPs have a tendency to aggregate. The theory behind this phenomenon is well-characterized [47,48]. Many groups use this as a method of detection, especially optical methods [49,50,51]. However, for the purposes of this design, aggregation is a source of error, since it can cause non-uniform binding on the surface. These results are particularly interesting as simulations did not consider the effects of this phenomenon on the impedance and sensitivity of our sensor. Simulations always assumed a uniform coverage of 10% GNPs, with 30 nm diameter [16]. However, GNP diameters naturally vary through random error and aggregation, which has a significant effect on its capacitance [45].

According to the impedance measurement graph in Figure 5d for a chip with large amounts of GNP aggregation, the maximum change from APTES modification to GNP modification is at a much lower frequency than with the previous results with more uniform GNP distribution (Figure 4). The GNP modification still always increases the impedance. A decreasing phase is typical; however, its shift to the right is inconsistent with other GNP modification tests. The decrease is still likely due to the double layer capacitance of the GNPs. Note that there is very little change at the middle and higher frequencies. Non-uniform surface modification can cause more variation in impedance between tests. From the Figure 5e, it can be seen that many GNPs aggregated near the electrodes. In these tests, gold IDEs were used, suggesting the possibility that metallic bonding between the two gold substances was affecting results.

**Figure 6:**
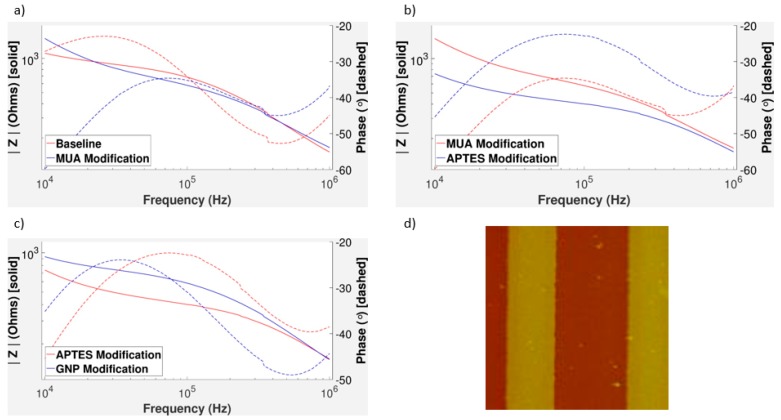
(**a**) Comparison between the baseline measurement, with no modification and the measurement taken after the electrodes were modified with MUA; (**b**) comparison between the APTES and MUA modification measurements; (**c**) the impedance measurement graph showing the change in impedance due to GNPs; (**d**) an image taken using an AFM to show the presence of GNPs, with less aggregation near or over the electrodes. Note that this chip was slightly over-etched during microfabrication so the dimensions of the electrodes are slightly off (10 μm × 10 μm area shown).

In an attempt to prevent clumping of GNPs on the electrodes, the possibility of a self-assembled monolayer was explored as data has shown that they can reduce corrosion [31]. We can see from Figure 6a that the addition of an extra layer over the gold IDEs leads to little to no change in the impedance magnitude. This is ideal, since a massive change in impedance magnitude from MUA could overshadow changes in GNP concentration. The capacitive effect of the layer, however, is noticeable, as we can clearly see the downward shift in the phase component of the impedance. Lack of interference between the electrode surface modification and the substrate modification is vital to produce similar results as we have seen with unmodified electrodes. From the impedance results, electrode modification does not appear to interrupt the effects of the spacing modification as indicated in Figure 6b,c. This graph matches the patterns of the graphs that were previously shown in the aluminum and gold results, where impedance magnitude increased between APTES and GNP measurements. The decrease in impedance after APTES modification also follows the trend shown in Figure 6c. However, perhaps most importantly, this limited the clumping of GNPs on the IDEs (refer to Figure 6d).

## 4. Discussion

We have characterized many surface modifications of our IDE chip design using both impedance measurement and an AFM. Electrode material, electrode modification, and substrate modification were all investigated. Impedance results showed that surface modification with APTES generally lowered the impedance magnitude over all frequencies. The adsorption of GNPs to the APTES binding sites increased the impedance through double layer capacitance. Tests were performed that show we can detect changes in concentration using GNPs. We found that aluminum IDEs acted similar to gold IDEs in impedance tests. They produced similar trends under the same surface modifications in comparison with themselves and simulation tests. While aluminum is both easier and cheaper to microfabricate electrode chips, gold has been shown to be more chemically stable, and will not interfere with many biological molecules [52,53]. It is also important to recognize that the electrode material can be changed based on the biological molecule being detected. This allows for greater adaptability and more opportunities in types of molecules that can be detected. Changes in substrate modification yielded consistent impedance changes, which allows for precise determination of changes in concentrations.

Possible sources of errors, such as oversaturation of APTES and GNP clumping were considered, and possible solutions were proposed. Errors in impedance results at each modification step showed how increased modification stabilized measurements. Optimization of the ideal frequency of a single impedance measurement for greatest sensitivity was explored and we found this frequency to be 85.7 kHz. AFM imaging revealed GNP aggregation appeared to occur more prominently near gold electrodes, likely due to the metallic bonding attraction between themselves.

The effects of modifying the surfaces of interdigitated electrodes in conjunction with the modified silicon substrate with the intent of preventing the aggregation of GNPs on the surface of the electrodes was explored. The electrical character of the chip surface becomes more capacitive as gold nanoparticles are bound between electrode digits. Aggregation of GNPs on the surfaces of the electrodes was shown to decrease compared to chips not modified with MUA. MUA was chosen to construct the self-assembled monolayer, while many other molecules have been shown to bind to gold [54], and their differing effects on impedance measurements requires further investigation. The electrode surfaces were modified only with single strands of MUA. Self-assembled monolayers can be polymerized [32,55], and the effects of such polymerization on impedance requires further research.

The ultimate goal is to use this biosensor to measure the presence and concentration of biological molecules, such as DNA, proteins, or metabolites. A sandwich assay can be used to attach GNPs to the surface; this can easily be modified to any available aptamers to detect any number of biological molecules. The only limitation on what can be detected is based on the availability and existence of aptamers for any given molecule. Due to the physical size of the chip and the microfabricated design, portability of the sensor is extremely feasible. Biological samples can be difficult to obtain and often can only in very small volumes. Our chip only required the addition of 50 μL solutions to the wells of the electrodes, allowing for opportunities to test very small bio-samples accurately. Due to the overall simplicity in design and miniaturization, costs of producing chips are relatively inexpensive compared to some other methods. Impedance tests can very easily be automated and programmed to work, allowing for users inexperienced with electrical circuitry to use this device such as nurses and doctors. This characteristic is vital for a point-of-care design. These characteristics of the chip allow for high versatility, for an inexpensive, portable, point-of-care biosensor device.

## 5. Conclusions

In this article, we discussed the techniques used to bind gold nanoparticles (GNPs) on interdigitated electrodes (IDEs), in addition to the characterization and detection of the surface modifications. Both measurement results and simulation results suggested that we can use impedance measurements to characterize surfaces modified with gold nanoparticles. Such an impedance-based design makes a versatile handheld, inexpensive biosensor device feasible.

## Figures and Tables

**Table 1:** The average impedance magnitude of the baseline test of four different wells at three different frequencies.

Baseline Tests	Average Impedance Magnitude (Ohms)	Standard Deviation (Ohms)	Coefficient of Variation
10 kHz	869.5	143.9	0.165
100 kHz	601.5	59.5	0.0989
1 MHz	194.2	30.8	0.159

**Table 2:** The average impedance magnitude of the impedance test after exposing the chip to oxygen plasma. This data is from the same four wells as Table 1.

Baseline Tests	Average Impedance Magnitude (Ohms)	Standard Deviation (Ohms)	Coefficient of Variation
10 kHz	735.9	103.9	0.141
100 kHz	562.6	53.7	0.0954
1 MHz	156.2	8.38	0.0536
